# Learning Local Texture and Global Frequency Clues for Face Forgery Detection

**DOI:** 10.3390/biomimetics10080480

**Published:** 2025-07-22

**Authors:** Xin Jin, Yuru Kou, Yuhao Xie, Yuying Zhao, Miss Laiha Mat Kiah, Qian Jiang, Wei Zhou

**Affiliations:** 1 Engineering Research Center of Cyberspace, Yunnan University, Kunming 650504, China; kouyuru@stu.ynu.edu.cn (Y.K.); yuhaoxie0322@163.com (Y.X.); zhaoyuying0909@gmail.com (Y.Z.); zwei@ynu.edu.cn (W.Z.); 2 School of Software, Yunnan University, Kunming 650504, China; jiangqian@ynu.edu.cn; 3 Faculty of Computer Science and Information Technology, Universiti Malaya, Kuala Lumpur 50603, Malaysia; misslaiha@um.edu.my

**Keywords:** face forgery detection, deepfake detection, bioinformatics, frequency domain, deep learning

## Abstract

In recent years, the rapid advancement of deep learning techniques has significantly propelled the development of face forgery methods, drawing considerable attention to face forgery detection. However, existing detection methods still struggle with generalization across different datasets and forgery techniques. In this work, we address this challenge by leveraging both local texture cues and global frequency domain information in a complementary manner to enhance the robustness of face forgery detection. Specifically, we introduce a local texture mining and enhancement module. The input image is segmented into patches and a subset is strategically masked, then texture enhanced. This joint masking and enhancement strategy forces the model to focus on generalizable localized texture traces, mitigates overfitting to specific identity features and enabling the model to capture more meaningful subtle traces of forgery. Additionally, we extract multi-scale frequency domain features from the face image using wavelet transform, thereby preserving various frequency domain characteristics of the image. And we propose an innovative frequency-domain processing strategy to adjust the contributions of different frequency-domain components through frequency-domain selection and dynamic weighting. This Facilitates the model’s ability to uncover frequency-domain inconsistencies across various global frequency layers. Furthermore, we propose an integrated framework that combines these two feature modalities, enhanced with spatial attention and channel attention mechanisms, to foster a synergistic effect. Extensive experiments conducted on several benchmark datasets demonstrate that the proposed technique demonstrates superior performance and generalization capabilities compared to existing methods.

## 1. Introduction

The rapid advancement of deep learning has significantly contributed to the evolution of face forgery technology. Modern face forgery methods are capable of generating highly realistic counterfeit face images, with the development of Generative Adversarial Networks (GANs) [1,2,3] and autoencoders [4,5] lowering the technical threshold for such forgeries. These advancements enable the batch generation of forged images, improving their quality to the point where they are often indistinguishable from genuine faces to the naked eye. As a result, the risk of misuse of face forgery has escalated, raising significant concerns about security. Consequently, the need for robust face forgery detection techniques has garnered substantial attention from both the research community and industry, aiming to address the security challenges posed by the widespread abuse of face forgery technologies.

**Figure 1:**
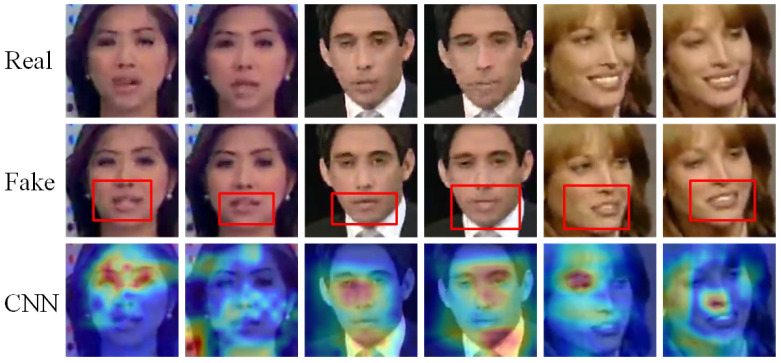
Example of Grad-Cam [9] of CNN network attention to face forgery. The CNN network is an EfficientNet [6]. The color shade reflects the attention level, the darker red gets more attention. The fake image is derived from the NT method in the FF++ dataset, and only the mouth region of the face is faked. The red box in the fake images plot roughly marks the faked region.

Face forgery detection involves distinguishing between real and fake facial images, where objective differences exist between the two. Current detection methods aim to learn these differences by training models on real and fake image datasets. Convolutional neural networks (CNNs) have become the predominant approach for face forgery detection, as illustrated in Figure 1. This figure shows the heat map of the region of interest generated by an EfficientNet [6] model applied to the FF++ [7] dataset of the NT [8] forgery method on a forged image. From the heatmap, we observe that the EfficientNet model exhibits two key limitations: firstly, it tends to focus too heavily on local regions, potentially overlooking other important forgery cues; secondly, the model fails to precisely locate the discrepancies between real and fake images. For example, the NT forgery method in the figure only alters the mouth region of the face, yet the model does not focus on this localized forgery. In fact, for some forged images, the model’s attention is mistakenly directed to non-facial regions. These issues underscore a fundamental challenge of CNN-based approaches, where misalignment in feature attention and the overfitting to localized patterns hinder the model’s ability to generalize across domains. Although CNNs achieve high performance within a dataset domain, they often struggle to maintain this effectiveness in cross-domain settings, leading to significant performance degradation. To address these challenges, we proposes a solution that enhances both the extraction of forged features and the global modeling capability of CNNs.

Early approaches to face forgery detection primarily relied on classification-based neural networks [6,10,11], where CNNs were trained to perform binary classification between real and forged images. Methods such as CapsuleNet [12] and MesoNet [13] introduced modifications to CNN architectures to enhance feature extraction. However, approaches that solely rely on CNNs often difficult to generalize when faced with previously unseen datasets or novel forgery techniques. To address this limitation, some methods [14,15,16] have explored physiological facial features, such as mouth movement during speech or blinking frequency, leveraging inter-frame variations in videos to detect forgeries. While effective in certain cases, these methods are limited by their dependence on specific facial gestures and motion cues, limiting their applicability to various types of forged media. Some studies focus on improving model learning by enhancing dataset diversity. Some methods [17,18,19] construct synthetic forgery images to enrich the variety of manipulated images or constrain the range of differences between real and fake images, allowing models to learn subtle and precise forgery patterns. However, these approaches demand substantial computational resources for data preprocessing. Moreover, the model’s performance remains highly dependent on the chosen forgery construction technique, making it challenging to adapt when confronted with unseen and more complex forgery datasets. Several image processing based techniques [20,21,22] have been proposed to improve detection. For instance, RFM [20] mitigates model sensitivity by masking specific regions, while DCL [21] leverages a combination of data processing techniques to construct contrastive learning models, demonstrating some improvement in cross-domain generalization. Recent work has also explored forgery detection across different domains. For example, GFF [23] employs Spatial Rich Model (SRM) [24] to extract high-frequency residual noise from images, while FreqNet [25] utilizes Fourier transform to detect inconsistencies in the frequency domain. These methods help mitigate overfitting to color information and expand the learnable feature space, thereby improving model robustness against diverse forgery techniques, but still underutilized features of different domains.

Drawing inspiration from biomimetic principles, we perform feature extraction and processing on forged images to achieve face forgery detection. First, inspired by the biological visual system’s response to and processing of image information at different spatial frequencies, we propose a comprehensive approach that utilizes both RGB color images and frequency-domain images for local and global image processing. This is further enhanced by wavelet decomposition for frequency-domain image processing, simulating the multi-scale characteristics of visual processing. Second, employing a CNN as the backbone network, whose architecture is inspired by the hierarchical and locally connected manner in which the animal visual cortex processes information, enables effective feature extraction and processing. Finally, referencing the human attentional processes of selective focus and information integration, we incorporate spatial and channel attention within the CNN. This promotes the synergistic utilization of features from multi-scale image inputs, thereby enhancing the model’s cross-scale modeling capability.

Inspired by the hierarchical information processing mechanisms of biological visual system, we use a CNN backbone. To enhance face forgery detection capabilities and mitigate the inherent limitations of CNN-based approaches, we introduce a novel framework that integrates advanced feature mining techniques with an optimized network architecture. Our goal is to improve CNNs’ ability to effectively extract forgery cues while addressing their tendency to focus excessively on localized regions. In the face forgery clues extraction stage, we extract local texture features and global frequency domain information. To prevent overfitting to high-level facial semantics, we propose a Local Texture Mining and Enhancement (LTME) module. This module extracts local texture information in segmented regions and selectively masks certain texture components to disrupt high-level semantic coherence. Consequently, LTME forces the model to focus on fine-grained forgery traces. Additionally, a texture enhancement submodule further amplifies these local features, guiding the model toward more precise forgery detection. For frequency domain feature extraction, the Global Frequency Feature Filtering Extraction (GFE) module leverages wavelet-based multi-layer frequency decomposition, inspired by the multi-scale information processing of biological vision systems. GFE strategically filters out low-frequency components that resemble the original image while preserving crucial global frequency information. This selective filtering reduces the model’s reliance on high-level semantic information that is independent of forgery artifacts, thereby improving its generalization ability. Following extraction, both local texture features and global frequency features are fed into the backbone network. To further enhance non-local modeling capability and effectively integrate local and global information, we propose a Local-Global Feature Enhancement (LGFE) module, drawing inspiration from biological attention processes. LGFE utilizes spatial and channel attention mechanisms to simulate the selective focusing and information integration processes of biological attention. Facilitating information exchange between local and global representations, thereby improving the model’s ability to capture forgery patterns across different scales and contexts.

Our contributions are summarized as follows:

- We propose a local texture mining and enhancement module (LTME) to extract forgery clues from local texture features and a global frequency feature filtering extraction module (GFE) to capture forgery cues from the frequency domain. These modules work together to reduce the influence of high-level semantic facial features, offering new approaches for detecting manipulation cues in face forgery images from both local and global perspectives.
- We propose a local and global feature enhancement module (LGFE) that enhances the model’s ability to synergistically exploit both local and global features. By employing spatial and channel attention mechanisms, the LGFE improves the model’s global perception and its capacity for non-local modeling.
- We conduct extensive ablation studies to validate the effectiveness of the proposed modules. In-domain comparison experiments and cross-domain experiments validate the model. Experimental results show that our method has high generalization ability while maintaining the highest accuracy.

## 2. Related Work

### 2.1. Conventional Face Forgery Detection

Early face forgery detection methods [6,10,12,13,26] simply regarded face forgery detection as a binary classification problem, using classification models to distinguish between real and face forgery images. However, these models were not specifically optimized for the unique characteristics of face forgery tasks. Due to the inherent limitations of convolutional neural networks (CNNs), such methods often overfit to local discrepancies within face forgery images, or even mistakenly focus on non-facial regions. While these approaches tend to perform well on in-domain datasets, they exhibit significant performance degradation when tested across domains. To address these limitations, several works have introduced more specialized methods [12,13,27]. For instance, Multitask Learning [27] employed a multi-task framework, utilizing an encoder-decoder architecture. The encoder performs binary classification, while a Y-decoder is used for region segmentation and input reconstruction, enabling simultaneous detection of forgeries and localization of manipulated regions. Nguyen et al. [12] propose Capsule Networks for face forgery detection. This method achieves comparable performance to traditional CNNs but with fewer parameters, making it more efficient. Furthermore, some studies [15,16,28] have explored biometric inconsistencies as clues for detecting face forgeries. For example, Agarwal et al. [28] detected video forgeries by analyzing the mouth shape during speech, while Yang et al. [15] exploited unnatural head poses as indicators of forgery in multimedia. Despite their success, these methods are constrained by the reliance on specific facial cues, limiting their generalizability to other types of face manipulations.

### 2.2. Uncovering Hidden Forgery Clues

To improve the generalization capability of face forgery detection models, recent studies [18,19,23,29,30,31] have focused on uncovering forgery traces that exhibit more robust and universal performance across various datasets. Some methods [17,18,19] rely on dataset processing techniques to self-construct forgery traces, enabling the model to learn a broader range of forgery types. For instance, SBI [19] segments the face from a single original image and reassembles it to create a forged image. Chen et al. [17] propose using generative adversary networks (GANs) [1] to perform forgery construction within a restricted region of the face. However, these methods often require significant computational resources for data pre-processing, and the effectiveness of forgery detection is heavily dependent on the specific dataset processing technique employed. Recent works [25,32,33] have sought to identify hidden, generic forgery cues across various domains of an image, such as the frequency domain or fine-grained texture features. Several studies have contributed to uncovering these subtle forgery patterns. For example, Sun et al. [21] used comparative learning between real and forged images to capture incongruent features. Zhu et al. [32] decomposed faces into 3D models and employed a dual-stream structure to highlight face details. Additionally, Luo et al. [23] and Fei et al. [34] utilized Spatial Rich Models (SRM) [24] filters to generate high-frequency noise modalities from RGB images. These noise features were then integrated into a dual-stream network to enhance the detection of forgery cues.

## 3. Proposed Method

**Figure 2:**
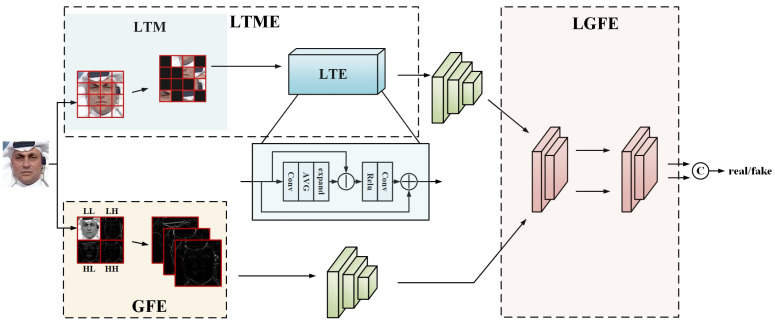
Overview of the proposed model. The LTME module consists of the LTM module and the LTE module, which are responsible for local texture feature mining and local texture feature enhancement, respectively. The green module is backbone, and we use EfficientNet-B4. The initial layer of backbone is processed and inserted into the LTM module for feature enhancement. The GFE module uses the wavelet transform for frequency domain feature extraction and rejects LL frequency features.

The overall architecture of the model is shown in Figure 2. The input color image is first processed in two ways to be used for local texture feature mining and global frequency domain feature mining respectively. For local texture feature extraction, we propose the local texture mining and enhancement module (LTME), which first divides the input image into patches, then masks a subset of these patches, and then re-combines these patches randomly as the input, and enhances the retained local information with features through the texture enhancement module. To extract global forgery traces, we propose the global frequency feature filtering extraction module (GFE), which applies a Discrete Wavelet Transform (DWT) to decompose the image into four distinct frequency components through two levels of wavelet decomposition. We remove the features most similar to the original image whose two decompositions are both low frequency, and retain the other global frequency domain features. Local and global features are feature extracted through the backbone network (EfficientNet [6]), and the local and global feature enhancement module (LGFE) is designed to enhance synergistic utilization of the two features in the model. The LTME, GFE, and LGFE modules are described in detail next.

### 3.1. Local Texture Mining and Enhancement Module

We avoid the learning of face identity information by the model by segmenting the face image into patches. As shown in Figure 2. Specifically, for any input image X, we first segment it into r×r patches, to conveniently match the model input resolution, we set *r* = 4. After segmentation, a random portion of the patches are masked, considering that there exists a region in the outermost part of the image that is not the location of the face, and contains less information than the central region of the patch, we mask the outer patches and the inner patches with different ratios. We randomly select 2(r−1) outer patches mask, and then mask the four corners of the outer patch. And in the inner layer ${\left(r-2\right)}^{2}/2$ patches are randomly selected for masking. That is, at least half of the outer patch is masked and half of the inner patch is masked. Finally, these patches are reorganized into $X^{\prime }$ in random order. Through the blocking and masking method, on the one hand, it destroys the overall structure of the face, avoids the influence of the high-level semantic information of the face (e.g., gender, appearance information) on the learning of the forged features, and highlights the local texture information of the face. On the other hand, it helps the model to avoid overfitting. After that, $X^{\prime }$ is input into the backbone, and we perform local texture enhancement after the initial layer of the backbone to enhance the low-level semantic texture features that are not yet abstracted by the backbone.

The process of local texture enhancement is shown in Figure 2 LTE. We first extract the feature $x^{\prime }$ from the initial layer output of the backbone by a set of 3×3 convolutions, and then perform an average pooling operation to capture the smoothed representation of the context to obtain the average feature $x_{a}^{\prime }$, after which we perform a bilinear interpolation process to adjust the scale of the average feature to the same scale as that of the feature before the average pooling, so that it can be used for subsequent operations. We subtract the two sets of features to obtain the salient texture residual information $x_{t}^{\prime }$ of the features, the process is as follows

(1) $$\begin{array}{c} x_{t}^{\prime }=x^{\prime }-x_{a}^{\prime }, \end{array}$$

Then the original features are augmented using the salient texture residual information, which is first processed using a bn layer and an activation function relu processing, and then the salient texture residual information is superimposed on the original features

(2) $$\begin{array}{c} x_{e}^{\prime }=x^{\prime }+x_{t}^{\prime }, \end{array}$$

The feature $x_{e}^{\prime }$ that finalizes the enhancement of the extracted texture is obtained, and the feature is used to lose back into the backbone to continue the feature extraction.

### 3.2. Global Frequency Feature Filtering Extraction Module

Methods of mining frequency domain information in image processing are diverse, such as mining image high-frequency residuals using a SRM [24], transforming frequency domain information using Fourier transform, and discrete wavelet transform [35,36]. Many face forgery detection methods try to mine image frequency domain modalities in the hope of finding traces of forgery hidden under the colors. From the perspective of avoiding model fitting to high-level semantic features of the face, we propose a global frequency feature filtering extraction module (GFE) based on discrete wavelet transform, which extracts global frequency domain features while extracting multiple frequency domain scale features through wavelet decomposition and excludes low-frequency features with high similarity to color images, so that the model focuses on finding more generalized features of the forged traces rather than the identity information of the face.

**Figure 3:**
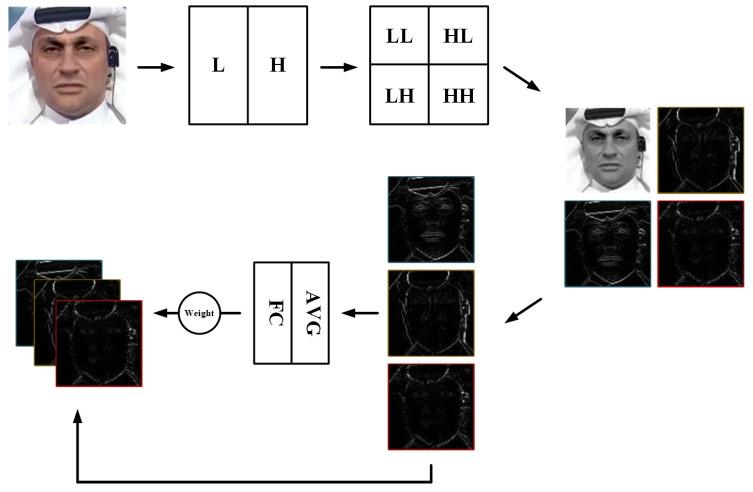
Illustration of GFE. The images are 2D discrete wavelet transformed and dynamically combined after removing LL.

We perform frequency domain feature extraction on the input image by means of a 2d discrete wavelet transform, as in Figure 3. Specifically, the image is decomposed by two wavelet filters using the Mallat algorithm [36]. For the four types of components obtained by decomposition: low frequency component (LL), horizontal detail coefficient (LH), vertical detail coefficient (HL), diagonal detail coefficient (HH). We do not use the low frequency component (LL) with the most face identity information. The 2d discrete wavelet transform performs two low pass filters on the image to obtain the LL image, which removes a large amount of high-frequency information and outputs low-frequency information. This LL image only loses the high frequency details compared with the original image, and has small differences with the original RGB image. To avoid feature duplication mining of frequency domain information and RGB texture information, we choose to use the rest of non-low-frequency components LH, HL and HH. For these components, we do not reconstruct the image together but reconstruct them into three image feature outputs respectively. And by dynamically assigning weights for feature fusion, the three frequency domain feature weights are adaptively weighted during training. Specifically, the feature importance is captured by global average pooling of the three image feature outputs, and then adaptive adjustment is achieved by dynamically generating weights for each sample through a layer of fully connected layers. We restrict the weights to a minimum value of 0.2, and use the softmax function to ensure that the sum of the weights is 1.

### 3.3. Local and Global Feature Enhancement Module

**Figure 4:**
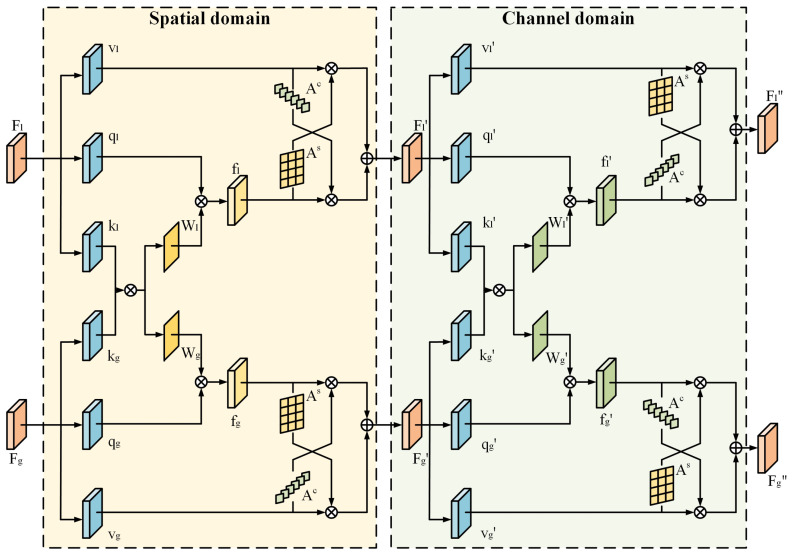
Illustration of LGFE. Enhancement and sharing of local and global features in the spatial and channel domains.

In order to make good use of local and global features, we design a local and global feature enhancement module (LGFE) to model the similarity of the two types of features and share feature weights to improve the synergy of the two types of features. The designed local and global feature sharing module is shown in Figure 4. The module consists of two primary parts. Spatial domain part models the interaction between local texture features and global frequency domain features through the cross-utilization of spatial attention. Channel domain part realizes the modeling of the relationship between the two types of feature maps through the sharing of weights by channel attention. By jointly leveraging spatial and channel-based feature interactions, the LGFE module enhances the model’s ability to synergistically utilize local and global features, thereby significantly improving the overall detection performance.

Specifically, the features extracted from the backbone are used as the initial inputs to the local and global feature sharing module, such that the local features are denoted as $F_{l}$ and the global features are denoted as $F_{g}$, and the subscripts l,g represent the two types of local and global features, respectively. The feature sharing enhancement is first performed from spatial domain by extracting the Query component, Key component and Value component from each of the two features: $q_{l}$, $k_{l}$, $v_{l}$ and $q_{g}$, $k_{g}$, $v_{g}$, where $q,v\in R^{C\times H\times W},k\in R^{C/r\times H\times W}$, the parameter *r* is a scalar used for dimensionality reduction of the channel to reduce the number of parameters and to improve the computational efficiency.The extraction of Key component is realized by two layers of convolution, the first layer of convolution with convolution kernel size of 3×3, which carries out the extraction independently, and the second layer of convolution with convolution kernel size of 1×1, and unlike the previous layer, this layer of convolution is shared for use during the extraction of both features, projecting the feature maps of both features into the same vector space. In addition, only one layer of convolution is used for Query component and Value component extraction. After that, the H and W in the Key component are spread out, at which point $k_{l,g}\in R^{C/r\times HW}$, and then the relevance weights $R_{s}\in R^{HW\times HW}$ of the two sets of Key components are computed by matrix multiplication, and the corresponding feature’s corresponding spatial relevance attentional weights $W_{s}\in R^{HW\times HW}$ are generated by the softmax function. spatial correlation attention weight $W_{l,g}$, the process is as follows:

(3) $$\begin{array}{cc} \; & R_{s}=k_{l}^{⊤}\times k_{g},\\ \; & W_{l}=softmax\left(R_{s}\right),\\ \; & W_{g}=softmax\left(R_{s}^{⊤}\right), \end{array}$$

The spatial correlation attention weights are matrix multiplied with the previously extracted Query component to obtain the spatially enhanced features ${f_{l}}^{\prime }$ and ${f_{g}}^{\prime }$. This spatially enhanced feature is then subjected to mutual enhancement operation with the previously extracted Value component $v_{l,g}$, specifically, the spatial attention map $A^{s}\in R^{1\times H\times W}$ is extracted from the spatially enhanced feature by two-layer 1×1 convolution, and the spatial attention map $A^{s}\in R^{1\times H\times W}$ is extracted from the spatially enhanced feature by global average pooling and two-layer 1×1 convolution to extract the channel attention map $A^{c}\in R^{C\times 1\times 1}$ from the Value component for cross-enhancement, i.e., using the spatial attention map for the Value component and the channel attention map for the spatially enhanced feature. The feature is then summed with the value component feature and channel merged by 1×1 convolution to complete the spatial domain feature enhancement, and the output features ${F_{l}}^{\prime }$ and ${F_{g}}^{\prime }$ are used as inputs for the subsequent shared enhancement of the channel domain features.

For channel enhancement, the local feature ${F_{l}}^{\prime }$ and global feature ${F_{g}}^{\prime }$ are also extracted with Query component, Key component and Value component to obtain ${q_{l,g}}^{\prime }$, ${k_{l,g}}^{\prime }$, ${v_{l,g}}^{\prime }$, where $q^{\prime },k^{\prime },v^{\prime }\in R^{C\times H\times W}$ component extraction operation is consistent with the component extraction operation for spatial domain. After that, the H and W in the Key components are also flatten, at which point ${k_{l,g}}^{\prime }\in R^{C\times HW}$, and then the correlation weights of the two sets of Key components ${R_{c}}^{\prime }\in R^{C\times C}$ are computed by matrix multiplication, and the corresponding channel correlation attentional weights for the corresponding features are generated by softmax function. The channel correlation attention weights ${W_{l,g}}^{\prime }$ of the features are generated by softmax function, which is the same as the component extraction operation of spatial domain, except that the order of the matrices is switched when matrix multiplication is used to extract the correlation weights ${R_{s}}^{\prime }$. Then the channel correlation attention weights are matrix multiplied with the Query components ${q_{l,g}}^{\prime }$ to obtain the channel enhancement features ${f_{l}}^{\prime \prime }$ and ${f_{g}}^{\prime \prime }$, and then mutual enhancement operation is performed. Then, the channel attention map $A^{c}\in R^{C\times 1\times 1}$ is extracted from the channel enhancement features, and the spatial attention map $A^{s}\in R^{1\times H\times W}$ is extracted from the Value component, and the two cross-enhancements are performed. Finally, the enhanced features are summed to obtain the features ${F_{l}}^{\prime \prime }$ and ${F_{g}}^{\prime \prime }$ that complete the enhancement of the local and global feature sharing modules.

### 3.4. Feature Fusion and Loss Function

We perform a connected combination of local and global features. Specifically, features extracted from the local stream and the global stream are first concatenated along the channel dimension. This concatenated feature map, encompassing both fine-grained details and holistic context, is then processed by a 1×1 convolution. The fused feature is subsequently passed through a ReLU activation function for non-linearity. This resulting fused feature vector, representing the combined local and global evidence, forms the input to the final classification stage. For the final classification decision, the fused feature vector is fed into a fully connected layer. This layer maps the high-dimensional fused features to a lower-dimensional space corresponding to the real or fake classes. Regarding the loss function, we use the cross-entropy loss function to minimize the discrepancy between the predicted probabilities and the ground-truth labels.

## 4. Experiments

### 4.1. Experimental Settings

#### 4.1.1. Datasets

To evaluate the effectiveness of our proposed method, we conduct experiments on three widely used benchmark datasets: FaceForensics++ (FF++) [7], Celeb-DF [37], and DeepFake Detection Challenge (DFDC) [38]. FaceForensics++ (FF++) is a widely adopted dataset that includes four types of face forgery techniques: Deepfakes (DF) [39], Face2Face (F2F) [40], FaceSwap (FS) [41], and NeuralTextures (NT) [8]. These methods can be categorized into two groups: Deep learning-based approaches, including Deepfakes and NeuralTextures, and Computer graphics-based methods, including Face2Face and FaceSwap. The dataset comprises 1000 real YouTube videos, each subjected to the four forgery techniques, producing a total of 4000 manipulated videos. FF++ provides videos at three resolution levels: raw, c23, and c40, with c23 being the most representative of real-world forgeries. In our experiments, we use the c23 version by default. Celeb-DF consists of 590 real videos collected from YouTube and 5639 high-quality Deepfake videos generated using an advanced Deepfake algorithm. It is available in two versions: Celeb-DFv1 and Celeb-DFv2, with Celeb-DFv2 being an improved and expanded version of v1. For our experiments, we use the Celeb-DFv2 dataset. DeepFake Detection Challenge (DFDC) is a large-scale Deepfake detection dataset introduced by Facebook (Meta). It includes videos manipulated using multiple, unknown forgery methods, making it a challenging benchmark for evaluating the generalization capability of forgery detection models.

#### 4.1.2. Evaluation Metrics

Following previous work, we employ two primary evaluation metrics: Accuracy (ACC) and Area Under the Receiver Operating Characteristic Curve (AUC). Accuracy (ACC) measures the proportion of correctly classified instances out of the total dataset, providing an overall assessment of the model’s correctness. Area Under the Curve (AUC), derived from the Receiver Operating Characteristic (ROC) curve, evaluates the classifier’s ability to distinguish between real and forged images. AUC represents the probability that the model ranks a randomly chosen positive instance higher than a randomly chosen negative instance. A higher AUC value indicates superior model performance, as it signifies better discrimination capability between genuine and forged samples.

#### 4.1.3. Implementation Details

We use EfficientNet-b4(Eff-b4) [6] as the backbone, which is initialized using the pre-training weights of ImageNet, and the input images are resized to match the backbone specification. To maintain consistency, this paper follows the FF++ dataset’s settings for face extraction methods and dataset partitioning, and 30 frames of face images are extracted for each video. The model is trained using stochastic gradient descent optimizer, with the initial learning rate set to $10^{-3}$ and weight decay set to $10^{-4}$. For the training parameters, the batch size is set to 32 and epoch to 15.

### 4.2. Comparison with Previous Methods

#### 4.2.1. In-Domain Evaluation

We conducted in-domain performance tests on the c23 compression version of the FF++ dataset and the Celeb-DF dataset. The FF++ (c23) version has the closest level of compression to the fake images that exist in real life. the Celeb-DF dataset has a high degree of realism that is indistinguishable by the human eye. As shown in Table 1, even though the existing methods have achieved high levels in the in-domain tests, our method achieves better results on both datasets. There is a 0.6% acc performance improvement and 0.35% auc performance improvement on the FF++ dataset. On Celeb-DF, a performance improvement of 0.09% in acc and a high level of auc are achieved. The GocNet [33], CLG [42], and PEL [29] methods in the table also extract the modalities of the images and construct network architectures with two branches, but the modalities are extracted differently from our method. GocNet labels residual traces by gradient operator, CLG extracts high-frequency features by SRM [24] filter, and PEL performs image processing using a discrete cosine transform. Compared with these similar methods, our method also shows superior performance, indicating that our feature extraction from the combination of local texture and global frequency domains can better utilize the characteristics of the two-branch network and synergize well to improve forgery detection.

#### 4.2.2. Cross-Domain Evaluation

To evaluate the generalization capability of our model, we set up two types of experiments. The first experiment involves training the model on a specific dataset and then testing it on other datasets to assess its ability to generalize across different datasets. The second experiment focuses on training the model on a dataset with a specific forgery method and subsequently testing its ability to detect authentic samples from datasets generated using other forgery methods. This setup allows us to evaluate the model’s performance in handling unknown forgery methods and its robustness in cross-method detection. The details of these experiments are as follows.

To evaluate the performance of the proposed method on unseen data, we trained our model on two compression degree versions, c23 and c40, of the FF++ dataset, and tested it on the Celeb-DF and DFDC datasets to assess its generalization performance across different datasets. These three datasets differ in terms of forgery methods and content characteristics, providing a robust evaluation of the generalizability of the method. As shown in Table 2, our method achieves excellent results on both the Celeb-DF and DFDC datasets across both compression levels (c23 and c40). The table indicates the specific compression degree version of the FF++ dataset used for training and records the backbone network utilized by each method, ensuring a comprehensive and fair comparison. From the table, we observe that our method performs optimally on the DFDC. For the Celeb-DF dataset, our method performs best in the FF++ (c23)-based training method, achieving a performance improvement of 0.24%, and also achieves good performance in the FF++ (c40)-based training method. These results demonstrate that our modal processing and feature extraction method excels at recognizing forged features and performs well in cross-dataset scenarios, highlighting its robustness and generalization ability for face forgery detection in previously unseen datasets.

To evaluate the performance of our method under unknown forgery generation techniques, we selected DeepFake (DF), a forgery method based on self-encoders, and Face2Face (F2F), a forgery method based on computer graphics, from the FF++ dataset for training. We then conducted generalization tests on each forgery method within the FF++ dataset. The results are presented in Table 3. For comparison, GFF [23] utilizes SRM filters to mine high-frequency noise features and constructs a two-stream network, while DCL [21] employs extensive data augmentation operations, including random patching. Both methods use EfficientNet-b4 as the backbone network. Compared to these approaches, our method outperforms most in various aspects. Specifically, the DF-trained network achieves a 10.13% performance improvement on NT, and demonstrates comparable performance on F2F, with a slight decrease in performance on FS. Additionally, the model trained using F2F shows 5.62% and 6.91% improvements on FS and NT, respectively, while maintaining competitive performance on DF, which highlights the effectiveness of our approach in extracting forgery features across different forgery methods. Overall, our method demonstrates superior generalization ability in scenarios involving unknown forgery generation techniques, outperforming other state-of-the-art feature mining methods in detecting face forgeries.

### 4.3. Ablation Study

We conducted a series of ablation experiments to assess the effectiveness of the individual components of our proposed method. These experiments primarily focus on two key aspects: modality extraction method and module architecture. For the feature extraction of different modalities, we performed ablation comparisons between our proposed local texture mining module (LTM) and other spatial domain processing techniques to evaluate the contribution of our approach to feature extraction in the spatial domain, and We compared our global frequency feature filtering extraction module (GFE) with other standard frequency domain information extraction techniques to examine the advantages of our approach in capturing high-frequency inconsistencies. For the module architecture, ablation experiments are carried out on the local texture enhancement module (LTE) as well as the LGFE module’s components that constitute the interactive utilization of local and global feature weights.

#### 4.3.1. Modality

As shown in Table 4, we constructed a variant network by employing different feature extraction methods to validate the effectiveness of our proposed localized texture extraction method (LTM) and global frequency feature filtering extraction module (GFE). Specifically, we replaced the spatial-domain and frequency-domain extraction methods to assess their individual contributions. In the frequency domain variant, we utilized commonly used methods, such as the SRM-based frequency domain residual extraction and the discrete wavelet transform (DWT), which share similarities with our approach. The other components of the model remained unchanged. The results show that our GFE module provides significant performance improvements, achieving 2.71% and 2.14% higher performance compared to the SRM and DWT methods, respectively. In the spatial domain variant, we tested different methods for processing color images, including an unprocessed RGB, as well as an approach involving only patching and random reorganization. The results highlight that our proposed LTM approach brings a substantial performance boost, particularly when compared to the unprocessed RGB. This demonstrates the effectiveness of our method in extracting masked face identity information and improving the model’s performance.

#### 4.3.2. Components

In the ablation experiments on the components within our model, we primarily focused on the local texture enhancement (LTE) module and the components of the local and global feature enhancement (LGFE) module. We constructed various model variants, as shown in Table 5. The results indicate that the best performance is achieved when all modules are incorporated. In (a1), high in-domain performance occurs, but the generalization is poor, possibly due to overfitting of the FF++ forgery feature. (a4) and (a5), which use only single-class attention enhancement, perform worse in both in-domain and generalization performance relative to our joint use of spatial and channel attention enhancement, suggesting that our approach is superior in exploiting the synergistic performance of the local texture and global frequency features. In addition, the generalization performance of (a4) shows some degradation relative to (a2), and the spatial enhancement reinforces the overfitting of the forged feature in the domain, which affects the generalization performance. These results highlight the effectiveness of texture enhancement and demonstrate that the weight sharing mechanism at both the spatial and channel levels between local and global features enables these two types of features to complement each other effectively. This synergy significantly improves the overall performance of the model.

### 4.4. Visualization

**Figure 5:**
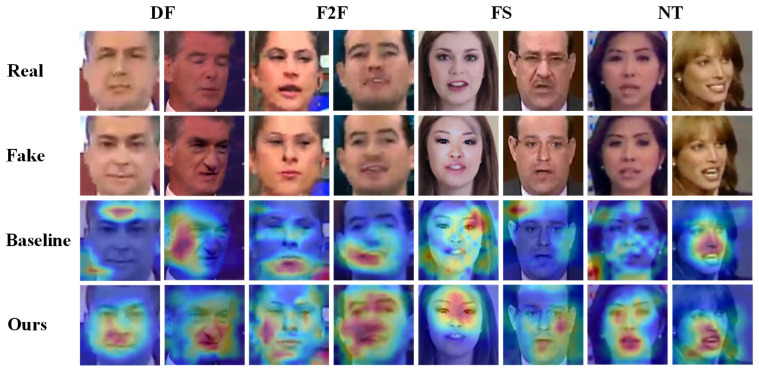
Examples of Grad-CAM [9] visualization face forgery detection. Warmer colors indicate higher model focus. The face forgery image is manufactured by NT [8], and NT performed mouth action forgery.

To explore the regions of interest identified by our proposed method, we utilized Grad-CAM [9] to generate heat maps, as shown in Figure 5. The heat map employs warm colors to indicate regions with high interest, where the intensity of the color corresponds to the level of attention the model places on those regions. The heat map is generated from forged images, with the corresponding real image from the neighboring frame provided in the first row for comparison. It is noteworthy that the NT forgery method is applied solely to the mouth region of the face. In the baseline Eff-b4 network, both the baseline and our method perform real or fake classification; however, the baseline primarily focuses on localized information, with some attention directed at non-forged areas or even non-facial regions. In contrast, our method successfully identifies forgery cues, with the region of interest typically centered on the forged areas within the face. This visualization underscores the effectiveness of our approach in extracting common forgery traces. It highlights the positive impact of our feature extraction method, which advances the semantic suppression of facial features, allowing the model to better capture forgery cues. Additionally, the synergistic use of local and global information enhances the model’s ability to accurately differentiate between real and fake images over a broader range.

## 5. Conclusions

In this work, we introduce a novel face forgery detection method that effectively mines forgery cues from both the color and frequency domains. We address the CNN’s excessive local attention to the non-forged parts of the face forgery image, and propose to perform local texture feature mining in the color domain and low-frequency filtering in the frequency domain to avoid the model’s overfitting to the non-forged semantics of the image, and to achieve the effective mining of the forged inconsistent cues. We perform spatial and channel attention sharing and enhancement between the RGB and frequency domains to establish the global attention of the model and realize the synergistic exploitation of the two types of features. Several experiments validate the effectiveness of our approach.

## Figures and Tables

**Table 1:** In-domain performances on FF++ and Celeb-DF.

Method	FF++		Celeb-DF	
	acc	auc	acc	auc
Xception [11]	95.73	96.30	97.90	99.73
Eff-b4 [6]	96.63	98.97	98.36	99.56
Meso4 [13]	93.10	-	-	-
F3Net [43]	97.52	98.10	95.95	98.93
Add-Net [44]	96.78	97.74	96.93	99.55
RFM [20]	97.60	99.29	95.69	98.79
RECCE [45]	97.06	99.32	98.59	**99.94**
MADD [26]	97.60	99.29	97.92	**99.94**
PEL [29]	97.63	99.32	-	-
GocNet [33]	94.34	97.75	-	-
CLG [42]	96.90	99.30	-	-
FSBI [46]	-	95.13	-	95.40
ID3 [47]	93.07	97.33	97.17	99.75
Ours	**98.23**	**99.67**	**98.68**	99.85

**Table 2:** Cross-domain evaluations on Celeb-DF and DFDC.

Method	Backbone	Train Set	Celeb-DF	DFDC
Xception [11]	Xception	FF++ (c23)	65.30	69.83
Eff-b4 [6]	Eff-b4	FF++ (c23)	68.52	70.53
Eff-b4 [6]	Eff-b4	FF++ (c40)	71.10	-
Meso4 [13]	-	FF++ (c23)	54.80	49.70
MesoInception4 [13]	-	FF++ (c23)	53.60	49.90
F3Net [43]	Xception	FF++ (c23)	65.17	66.90
Two Branch [48]	DenseNet [49]	FF++ (c40)	**73.41**	-
Face X-ray [50]	HRNet [51]	FF++ (c23)	74.20	71.20
RECCE [45]	Xception	FF++(c40)	68.71	-
GocNet [33]	ResNet [10]	FF++ (c40)	59.46	-
GocNet [33]	ResNet [10]	FF++ (c23)	67.43	-
GFF [23]	Eff-b4	FF++ (c23)	65.20	-
PEL [29]	Eff-b4	FF++ (c40)	69.18	63.30
MADD [26]	Eff-b4	FF++ (c23)	67.44	-
PFG [52]	Eff-b3	FF++ (c23)	75.32	-
MLPN [53]	Autoencoder	FF++ (c23)	62.49	61.55
ID3 [47]	Eff-b4	FF++ (c23)	71.18	65.86
Ours	Eff-b4	FF++ (c40)	72.16	**73.47**
Ours	Eff-b4	FF++ (c23)	**75.56**	**76.34**

**Table 3:** Cross-domain evaluations on FF++.

Method	Train Set	Test Set			
		DF	F2F	FS	NT
Eff-b4 [6]	DF	99.53	69.91	49.54	75.68
GFF [23]		99.87	76.89	47.21	72.88
DCL [21]		**99.98**	77.13	**61.01**	75.01
Ours		99.85	**77.34**	46.82	**85.14**
Eff-b4 [6]	F2F	84.52	99.20	58.14	63.71
GFF [23]		89.23	99.10	61.30	64.77
DCL [21]		**91.91**	99.21	59.58	66.67
Ours		91.83	**99.87**	**65.20**	**73.58**

**Table 4:** Ablation study of feature extraction strategy.

Method	Feature Type	Celeb-DF
SRM	frequency domain	72.85
DWT		73.42
Ours		**75.56**
RGB	spatial domain	70.65
Random patch		74.77
Ours		**75.56**

**Table 5:** Ablation study of different components. “✔” indicates the component is contained. FF++ is an in-domain test with ACC. Celeb-DF is a generalizability test with AUC.

ID	LTE	Spatial Enhance	Channel Enhance	FF++	Celeb-DF
(a1)				**98.66**	72.68
(a2)	✔			97.67	73.46
(a3)		✔	✔	98.41	74.54
(a4)	✔	✔		97.94	73.10
(a5)	✔		✔	98.08	74.51
(a6)	✔	✔	✔	98.23	**75.56**

## Data Availability

Data availability online: https://github.com/ondyari/FaceForensics (accessed on 31 July 2022), https://github.com/yuezunli/celeb-deepfakeforensics (accessed on 5 December 2022) and https://www.kaggle.com/c/deepfake-detection-challenge/data (accessed on 5 December 2022).
